# Towards Robustifying Image Classifiers against the Perils of Adversarial Attacks on Artificial Intelligence Systems

**DOI:** 10.3390/s22186905

**Published:** 2022-09-13

**Authors:** Theodora Anastasiou, Sophia Karagiorgou, Petros Petrou, Dimitrios Papamartzivanos, Thanassis Giannetsos, Georgia Tsirigotaki, Jelle Keizer

**Affiliations:** 1 UBITECH Ltd., Thessalias 8 and Etolias 10, GR-15231 Chalandri, Greece; 2 Hellenic Army Information Technology Support Center, 227-231, Mesogeion Ave., GR-15451 Holargos, Greece; 3 Philips, Oliemolenstraat 5, 9203 ZN Drachten, The Netherlands

**Keywords:** adversarial machine learning, adversarial training, AI security

## Abstract

Adversarial machine learning (AML) is a class of data manipulation techniques that cause alterations in the behavior of artificial intelligence (AI) systems while going unnoticed by humans. These alterations can cause serious vulnerabilities to mission-critical AI-enabled applications. This work introduces an AI architecture augmented with adversarial examples and defense algorithms to safeguard, secure, and make more reliable AI systems. This can be conducted by robustifying deep neural network (DNN) classifiers and explicitly focusing on the specific case of convolutional neural networks (CNNs) used in non-trivial manufacturing environments prone to noise, vibrations, and errors when capturing and transferring data. The proposed architecture enables the imitation of the interplay between the attacker and a defender based on the deployment and cross-evaluation of adversarial and defense strategies. The AI architecture enables (i) the creation and usage of *adversarial examples* in the training process, which robustify the accuracy of CNNs, (ii) the evaluation of *defense algorithms* to recover the classifiers’ accuracy, and (iii) the provision of a *multiclass discriminator* to distinguish and report on non-attacked and attacked data. The experimental results show promising results in a hybrid solution combining the defense algorithms and the multiclass discriminator in an effort to revitalize the attacked base models and robustify the DNN classifiers. The proposed architecture is ratified in the context of a real manufacturing environment utilizing datasets stemming from the actual production lines.

## 1. Introduction

Image classification algorithms fueled by artificial intelligence (AI) and realized via deep neural network (DNN) architectures are commonly used into several application domains for image recognition and classification [1], object detection [2], and image retrieval [3]. These algorithms are trained over many image examples to create mathematical representations or statistical models to predict with high accuracy whether the pixels of a new image are more likely with the set of classes/categories under examination. In fact, AI-based image processing and recognition are not just methods evolved in research papers and evaluated in lab environments. Those barriers were broken a long time ago, and nowadays, AI-based image processing and recognition constitutes the core of critical applications of high technology readiness levels, leaving a wide footprint on industrial applications.

Among others, Industry 4.0, which will be the focal point of the evaluation of this work, capitalizes on AI methods to enable flexible production lines and support innovative functionalities such as mass customization, predictive maintenance, zero-defect manufacturing, and digital twins. Large-scale AI deployments in manufacturing involve many interactions between the AI systems and other elements of the surrounding environment, including hardware, software, and physical systems. Thus, in order to ensure the success of this blend of technologies, the design of trusted and reliable AI systems positioned in the manufacturing floors is of the utmost importance.

On the downside, the emergence of adversarial machine learning (AML) has become a major concern towards the adoption of AI technologies for critical applications and has already been identified as a barrier in multiple application domains. AML is a class of data manipulation techniques that cause changes in the behavior of AI algorithms while, usually, going unnoticed by humans. An ever-increasing problem is that AML can create slight modifications over the mathematical representations and, therefore, over the model, which may remain imperceptible to the human eye while changing the output of an AI system. Suspicious object misclassification in airport control systems [4], computer vision systems of autonomous vehicles resulting in moving into the opposite traffic lane [5,6], tricking healthcare image analysis systems into classifying a benign tumor as malignant [7,8], and abnormal robotic navigation control [9] in the context of human-robot collaboration setups are only a few examples of AI models’ compromise that advocate the need for the investigation and development of robust defense solutions.

The challenges posed by AML have recently attracted the attention of the research community, the Industry 4.0, and the manufacturing domains [10,11] as possible security issues on AI systems which can pose a threat to systems reliability, productivity, and safety [12]. The densely interconnected systems of the modern manufacturing floors in conjunction with their transition to open networks give room to adversaries to infiltrate into the factories’ ICT networks by exploiting vulnerable services. In this context, DNN-based systems become a potential target of advanced adversaries that may employ different attack tactics and techniques for compromising the operation of a DNN either by taking control over the system or by altering the input data in a way that outputs malicious decisions. For example, an adversary can attack an AI system to access confidential data or proprietary learning models that could lead to intellectual property (IP) theft. By having access to systems, an adversary can compromise the data used for training an AI system or even disclose the rules of an AI-based operation used for decision making. These attacks can lead to IP theft while also compromising the proper operation of AI systems that may introduce risks in the production processes or degrade their benefits. Beyond cyber security attacks, data unreliability can be caused by other factors such as data transfer errors, signal distortion, communication channel interference, and more. Unreliable data represent one of the major challenges for the graceful operation of AI systems, as such data can lead to biased and erroneous AI applications and thus decisions.

In this reality, defenders are not just passive spectators. On the other side of the same coin, AML defense algorithms have made significant progress in uncovering the space of AML, providing solutions that act both on the digital domain (i.e., AI-based digital services and system operations) and even on more recent cases which are applicable to the physical dimensions [13] considering emerging physical systems using DNNs in safety-critical situations. The emergence of AML has led the community to the systematic documentation of adversary tactics, techniques, and case studies for AI systems based on real-world observations (e.g., MITRE ATLAS [14]) in an effort to raise awareness of these threats and form a common understanding among security researchers and professionals. The most prominent attack techniques may occur in both the training (i.e., poisoning) and the operational (i.e., evasion) phases of DNNs.

In this context, there is a pressing need for robustifying image classifiers against diverse perils of adversarial attacks against AI systems. Research needs to be intensified and drill into the details and the specificities of existing AI systems, investigate their vulnerabilities, and introduce solutions that make DNNs more resilient against adversarial inputs, attempts of poisoning, and evasion attacks.

In this paper, we introduce a robust AI architecture empowered with multiple AML attacks and defense algorithms using the open-source Adversarial Robustness Toolbox (ART) [15]. The toolbox supports multiple programmable methods to defend and evaluate the proposed architecture against the adversarial threats of evasion and poisoning of a real-world AI system coming from the manufacturing domain. The AI architecture implements a preprocessing module for image curation and cleaning before the AML attacks. A *multiclass discriminator*, as a post-processing module, generates alerts and reports on the detection of evasion attempts. The proposed architecture and the individual attacks and defenses are evaluated in the context of a real manufacturing environment, utilizing real industrial datasets stemming from the DNN-based Visual Quality Inspection system deployed in Philips’ factory. Our evaluation results advocate that the proposed approach offers advanced resilience and recovery to the quality inspection system by augmenting the training dataset with adversarial examples and by training the multiclass discriminator in order to distinguish between non-attacked and attacked classes of the datasets. In addition, the designed evaluation testbed gives the opportunity to investigate the behavior of attack-defense pairs in order to conclude to the most appropriate defense which can deliver a more robust AI system based on DNN models, which, in this specific case, are built on convolutional neural network (CNN) classifiers.

Overall, the contributions of this paper are as follows:

- We introduce a reproducible AI architecture for robustifying CNN image classifiers that has been evaluated in the context of a real manufacturing environment which is prone to noise, vibrations, and errors when capturing and transferring data.
- We present a thorough evaluation of attack-defense pairs for uncovering the peculiarities of different adversarial techniques and investigating the applicability and effectiveness of the defense strategies under real conditions.
- We deliver a modular approach for the recruitment of different modules (i.e., a *preprocessing module*) and techniques to robustify and guide the deployment of AML attacks and defense algorithms (i.e., via the creation of *adversarial examples* augmented in the model’s training phase and *defense algorithms*) for revitalizing CNN classifiers’ accuracy and detection (i.e., through the *multiclass discriminator*).
- We document a generic and applicable solution which adapts to domains dealing with computer vision and image processing applications using CNN classifiers.

The rest of this paper is organized as follows. In Section 2, we present in a grouped manner different techniques and how these address the problem of AML attacks. These techniques have been selected because they are closely related to the hybrid but combinatorial solution we introduce in this work. In Section 3, we present the datasets, the manufacturing ecosystem and the usage of the proposed solution in real settings. In Section 4, we present the proposed AI-based quality inspection architecture along with its software modules. Section 5 outlines the evaluation methodology and describes the experimentation results. Finally, Section 6 provides an overall discussion on the findings and concludes.

## 2. Related Work

Adversarial machine learning attacks are considered a severe threat to AI systems since they can easily change their output using different manipulation techniques. These circumstances necessarily force the research community to devise new robust and resilient methods to safeguard AI systems. Therefore, this section will briefly cover the most relevant and up-to-date research works, mostly related to robustifying image classifiers and enforcing defensive strategies against the perils of adversarial attacks. The literature review shows that either denoising the image classifiers, training the target model with adversarial examples, known as *adversarial training*, or applying defense algorithms is a common countermeasure to tackle AML attacks.

Akhtar et al. [16,17] presented a comprehensive study in this area including the first generation of AML attacks and their defenses in computer vision and machine learning (ML) research. Additionally, Kloukiniotis et al. [5] investigated strategies for robustifying scene analysis of adversarial road scenes. Through their review, they introduced a taxonomy of the defense mechanisms for countering adversarial perturbations by classifying those mechanisms into three major categories: those that modify the data, those that propose adding extra models, and those that focus on modifying the models deployed for scene analysis.

Denoising image classifiers is an approach currently adopted to address the problem of AML attacks. Specifically, Yan et al. [18] investigated the adversarial robustness of deep image denoisers. They studied how well they can recover the original information from noisy degraded observations. For this reason, a novel adversarial attack called observation-based zero-mean attack was introduced to evaluate the robustness of the proposed deep image denoisers. Moreover, a hybrid adversarial training strategy was proposed to ensure the quality of the reconstructed information. Similarly, Pawlicki et al. [19] proposed some preprocessing defenses, including a block-matching CNN for image denoising. The advantages of such defenses include the absence of retraining the classifier, which usually, in computer vision problems, is a demanding and computationally heavy task. Furthermore, spiking neural networks (SNNs) can be easily implemented on neuromorphic chips providing energy-efficient learning capabilities with event-based dynamic vision sensors (DVS). The robustness of SNNs against adversarial attacks on such DVS-based signals by using noise filters for such sensors has been studied by Marchisio et al. [20]. The results were promising, and they prevented SNNs from misleading identifications. Liu et al. [21] proposed new ways of investigating the robustness of CNNs, widely used for image segmentation.

In addition to the approaches mentioned above, training the target model with adversarial examples is another countermeasure to AML attacks [22]. The effectiveness of adversarial training via data augmentation and distillation has also been studied to tackle adversarial attacks efficiently. Specifically, Bortsova et al. [23] focused on adversarial black-box settings, in which another AI model is usually used for the target model. The effects of weight alterations and the differences while developing target and surrogate models have been thoroughly studied. Hashemi and Mozaffari [24] trained CNNs with perturbed samples manipulated by various transformations and contaminated by different noises to foster the robustness of neural networks against adversarial attacks.

Finally, applying defense algorithms is another strategy to tackle AML attacks. Xi [25] discussed three main categories of attacks against AI systems. Then, she introduced some defense approaches to tackle the problem. A coherent benchmark to evaluate adversarial robustness on image classification tasks has been proposed by Dong et al. [26]. In this work, large-scale experiments with two robustness curves have been conducted. Kyrkou et al. [27] proposed a cyber threat detection system called CARAMEL. The system offers many detection techniques to combat incoming security risks in automotive driving systems with embedded camera sensors. Moreover, since the solutions developed by CARAMEL are lightweight and low power, they can be easily hosted on energy constraint processors and platforms, offering additional robustness and energy efficiency.

In reality, defenders of AI systems are uninformed about the tactics and mechanisms that are applied by the attackers. Therefore, it is pivotal to utilize hybrid but combinatorial approaches and more focused alternatives to improve AI systems’ robustness. Motivated by the limited number of research works in this area, we introduce a *hybrid approach against AML attacks*. Our approach uses input transformation operations as preprocessing, generates adversarial examples to augment with noise the input dataset, and evaluates the robustness of the AI system under several adversarial attack setups and defense algorithms for recovery.

## 3. Datasets and Manufacturing Ecosystem

We briefly present the datasets, the manufacturing ecosystem and the usage scenarios to let the reader follow different references about them across the paper.

### 3.1. Dataset Description

Each dataset used in this study has its own characteristics and specificities. To preserve business-related details, we refer to them as *Dataset_1* and *Dataset_2*.

#### 3.1.1. Images from *Dataset_1*

**Figure 1:**
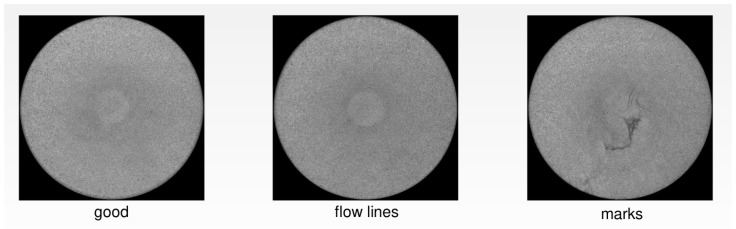
Variability of *Dataset_1*.

*Dataset_1* contains images of a decorative part of the Philips shavers that are produced in high volume. Since Philips maintains the highest standards regarding the visual appearance of produced products, 100% quality inspection of these parts is required to guarantee perfect quality. *Dataset_1* has 592 samples, of which 203 are normal samples, 198 are samples with flow lines, and 191 are samples with marks, as presented in Figure 1.

#### 3.1.2. Images from *Dataset_2*

**Figure 2:**
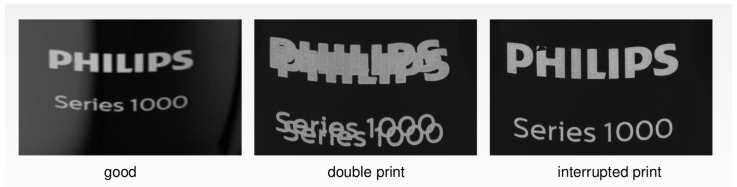
Variability of *Dataset_2*.

*Dataset_2* contains images of the printed Philips logos on Philips products. This dataset is limited to the prints of one product type. The visual inspections of these parts is crucial to ensure that only perfect quality is shipped from the factory to the different customers. *Dataset_2* has 2700 normal samples, 244 double-print samples, and 620 interrupted-print samples, as presented in Figure 2.

For example *Dataset_2*, in order to support a classification task, has been separated into three subcategories, e.g., good, double-print and interrupted-print. The proposed data-centric solution aims to build a deep neural network architecture that predicts to which subcategory a new image from the application field belongs. The architecture also enables to distinguish the three original subcategories and the three attacked subcategories generated via the *adversarial imagery examples* and reports on their category, e.g., attacked vs. non-attacked and more.

As aforementioned, each dataset has its own characteristics. In fact, this is also reflected in the number of instances of each class of a dataset. As can be noticed, *Dataset_1* is more balanced than *Dataset_2*. This is actually a strategic choice in the context of our evaluation testbed, as the distributional characteristics of the datasets reflect the actual conditions met in the production lines of the manufacturing environment. Based on our experience on the behavior of the manufacturing process and the quality inspection system in place, the datasets constitute representative samples of data captured for a given duration of the production line operation. Of course, the dataset sample distributions may affect the behavior of the trained algorithms, especially for the case of the unbalanced *Dataset_2*. However, it is important to convey this balance in order to stress test the adversarial concept, consider realistic and aggressive AI attack strategies and design defenses as close as possible to realistic conditions.

**Figure 3:**
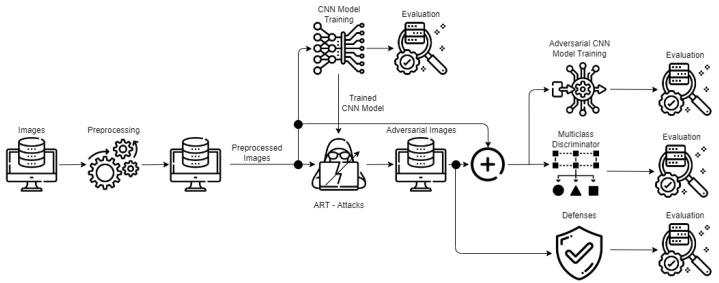
AI-based quality inspection architecture.

The data-centric AI architecture of Figure 3 performs a preprocessing step that helps to clean, filter, resize and extract the essential features from the images. Then, in the proceeding step, we train a *base CNN model* as a baseline and evaluate its accuracy to investigate how much the latter is affected by different ART attacks [15]. We also want to see the contribution to generating *adversarial examples* augmented in the *adversarial training* of the base CNN model and at which percentage its accuracy can recover by applying different *defense algorithms*. The *multiclass discriminator* is trained to distinguish between attacked and non-attacked images and their categories. The following sections present more details about the different modules and scenarios to assess the data-centric AI architecture.

### 3.2. Manufacturing Ecosystem and Threat Model

The Philips factory in Drachten, the Netherlands, is an advanced factory for the mass manufacturing of consumer goods (e.g., shavers, OneBlade, baby bottles, and soothers). Current production lines are often tailored for the mass production of one product or product series in the most efficient way. However, the manufacturing landscape is changing. Due to global shortages, manufacturing assets and components are becoming scarcer, and a shift in market demand requires the production of smaller batches more often. To adhere to these changes, production flexibility, re-use of assets, and a reduction of reconfiguration times are becoming more important for the cost-efficient production of consumer goods. In this context, one of the topics currently investigated within Philips is the painless setting up of the automated AI-based quality inspections that aim to make the reconfiguring of quality control systems faster and easier.

**Figure 4:**
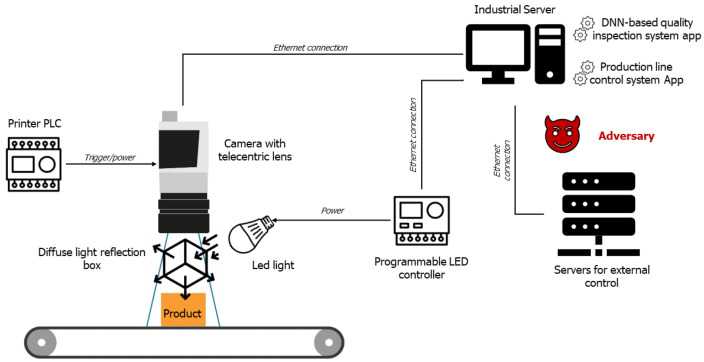
Manufacturing ecosystem and adversarial threat model.

The setup used to inspect the quality of the printed logo on the Philips products can be seen in Figure 4. In this setup, the printer will trigger the visual quality control system whenever a print has been applied upon a product. This system comprises a few different assets, including a camera, lighting and a light reflection box. The lighting combined with the box ensures that the image taken from the product is visible and creates a consistent environment for the quality control algorithm to determine whether a product is of good quality or not. This decision is made by a quality control algorithm running on the industrial server based on the DNN-based system destined to classify the images captured by the inspection camera. More specifically, the system is based on a convolutional neural network (CNN) to perform pattern recognition and classification over visual imagery. The CNN-based classifier has been trained based on manually collected historical data reflecting all possible flaws generated by the automated manufacturing line. During production time, the captured images are given as input to the pre-trained CNN-based quality inspection system to perform the necessary checks on the quality of the product. Based on the decision taken by the latter app, the product will continue its journey to the factory’s assembly lines, or it will be discarded. One can easily understand that the quality inspection system is a mission-critical asset in the production workflow to ensure the high quality of the end products and avoid the undesirable assembly of faulty parts. This is also translated into increased production cost and the waste of essential time due to unnecessary machine engagement, slowing down the production rates.

The DNN-based quality inspection is based on a classic machine learning model setup. Its training takes place on the servers for external control, under human supervision. The historical datasets reside on the file system of the external server and have been manually gathered and stored. The human has the necessary ML domain and application knowledge to carry out the experimentation and the classification model training. The resulting model is then placed manually in the file system of the industrial server, and the CNN-based QI app puts the model in the inspection workflow.

**Evasion Attacks:** In this work, we consider adversaries, under the notion of ML model evasion attacks [28], who can craft adversarial data which can lead a machine learning model to identify the contents of the data incorrectly. This technique can be used to evade a downstream task where machine learning is utilized to empower a detection system. In this context, the adversary tries to manipulate systems and data and evade the model during the inference mode. For instance, the adversary may exploit a vulnerability on the visual inspection camera and compromise the integrity of the captured data by manipulating this business resource operational behavior. Under this adversarial approach, the corresponding business processes can look fine, but may have been altered to benefit the adversaries’ goals.

**ML Attack Staging:** In addition, we consider an adversary who may attempt to poison the target model and craft adversarial data [29] to feed the DNN model and obtain the intended result. This approach will enable the adversary to create “vulnerable” trained models using data that may not be easily detectable during the training phase. The injected vulnerability can be activated at a later time by injecting poisoned data samples in the testing phase after the adversary gains access to the system (e.g., by exploiting a remote access vulnerability of the targeted systems).

In this context, the attacks and defenses generated and evaluated in Section 5 consider the aforementioned adversary capabilities and proceed to the evaluation of defensive strategies to conclude with the most robust setup in the context of the above-mentioned QI setup. Our aim is to identify the model and strategy that will be interposed in the dataflows of the training or the testing phases to try to sanitize the data pipelines by filtering out malicious instances or by detecting the injection of adversarial examples in the process. Hence, we assume that the attacker can gain access to the environment’s resources by exploiting system vulnerabilities that enable her to gain access to critical systems or inject the malicious samples remotely, and thus, to interfere with the DNN-based QI model. Thus, we assume that the attacker can target the industrial servers, the server for external control, or the inspection camera per se.

### 3.3. Usage of the AI-Based Quality Inspection Solution

The generation and augmentation of adversarial examples (i.e., *adversarial training*) while training an AI model to boost its resilience is a common defense strategy to let the latter classify and predict more enhanced features. While the adversarial examples are useful for the purposes of augmenting benign datasets, there is an imminent need for frameworks that enable the exploration of realistic adversarial attacks with varying threat models. For instance, in our case, we define realistic attacks for image classification tasks involved in an AI-based quality inspection solution in a shop-floor. This enables early decision-making before the product is released to the next step of the production line, i.e., right after the label printing. Attacks may come in at different stages. For example, digital attacks such as the projected gradient descent (PGD) are injected at the recognition stage. In Section 5, we present in detail a comparison among varying methods of attacks and defenses captured by the proposed architecture as part of a quality inspection solution.

## 4. Logical Modules and AI-Based Quality Inspection Architecture

This paper considers a data-centric AI architecture that is applied for quality inspection (QI) in a shop-floor production line. Figure 3 presents how the data travel and are modified through the proposed architecture.

The logical modules of the architecture are the following:

- *A preprocessing module*, which is responsible for images curation, cleaning and performs resizing and filtering;
- *Training and evaluation of the base CNN model*, which serves as the baseline for the evaluation of the model’s accuracy before and after the attacks and defenses;
- *Adversarial imagery example generation and adversarial CNN model training*, which takes as input the preprocessed images, passes them through different ART attacks to modify them to generate adversarial imagery examples, and then trains an *adversarial CNN model*;
- *A multiclass discriminator*, which combines the preprocessed images and their labels/categories along with the adversarial imagery examples with their labels/categories. We build a multiclass classifier which lets the model distinguish images between attacked and their category vs. non-attacked and their category;
- *Defense algorithms*, which enforce methods enabling the recovery of the attacked images and raise the accuracy of the base CNN model.

For this study, we analyzed and processed datasets coming from a shop-floor production line at the level of quality inspection. This step of quality inspection is critical to the business of manufacturing because it determines if the product will be propagated to the finalization stage, packaging, and route to market or will be withdrawn.

### 4.1. Preprocessing Module

The *preprocessing module* is necessary to curate the images in order to let more features be extracted during the training of the models. The module has been implemented using Keras as the preprocessing library [30]. After data visualization and exploration, we applied filtering, resizing, and data normalization under a specific numeric range. We then prepared and split the images for multiclass or binary classification. The multiclass classification predicts the image category and whether it has been attacked, while the binary classification only distinguishes between attacked (i.e., malicious) and non-attacked (i.e., usual) images.

*Dataset_1* is natively challenging to distinguish the differences in two out of three categories because it has good and flow lines, rendering the samples not trivial to process and extract features due to their close similarity. *Dataset_2* is easier to handle for feature extraction, as presented in the following paragraphs.

**Figure 5:**
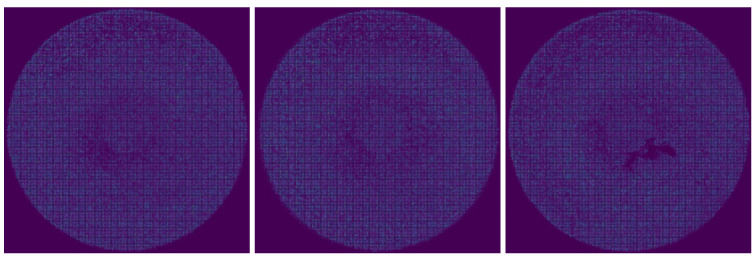
Histogram of oriented gradients applied to *Dataset_1* in order to extract useful information and disregard the unnecessary information from the image.

More specifically, for *Dataset_1*, we took the following steps. The images were loaded from the local database and transformed to arrays by using the Keras library. Next, we applied some filtering methods to clean and make the dataset more visible. More specifically, we used exposure filters and the histogram of oriented gradients (HOG) technique [31]. HOG is a feature extraction method primarily used in computer vision and image processing applications for object detection. This method counts events of gradient orientation in a specific portion of an image or a region of interest that we choose. Finally, we normalized the images arrays within the range [0,1]. An example of applying the HOG technique over *Dataset_1* to extract more features is depicted in Figure 5.

**Figure 6:**
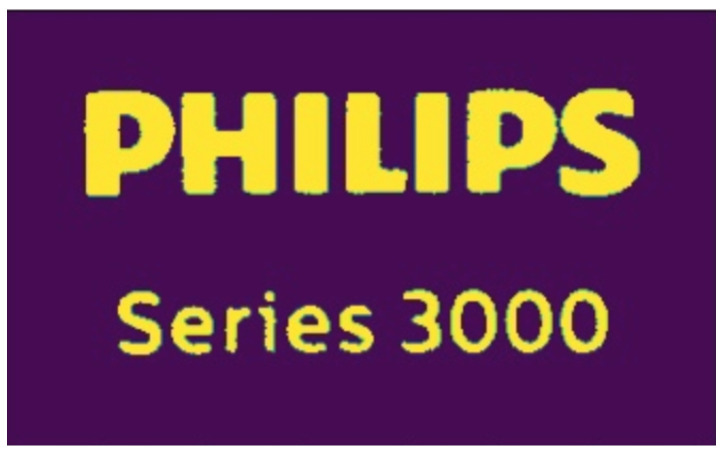
Otsu’s threshold applied to *Dataset_2* to apply image thresholding for image binarization based on pixel intensities and contribute to better pattern recognition.

The images of *Dataset_2* were loaded from the local database and transformed into arrays using the Keras library. In addition, to ensure that all the features are visible and can be extracted to train the models, we applied Otsu’s threshold [32], which has the ability to filter features out of the images. Next, a normalization step was followed in the range of [0,1]. An example of applying the Otsu’s thresholding method over *Dataset_2* to extract more features is depicted in Figure 6.

### 4.2. Training and Evaluation of the Base CNN Model

The training and evaluation of the *base CNN model* facilities to build a model which serves as the baseline to investigate its robustness measured by means of accuracy under two concrete conditions: (i) after applying different attack methods by using the ART tool [15]; and (ii) after applying different defense algorithms to quantify at which percentage the accuracy of the *base CNN model* can recover from the attacks. Table 1 presents the configuration parameters. The same model is then fed to the ART tool [15] by performing different attacks in order to generate the *adversarial imagery examples*. To build the *base CNN model*, we used different Keras layers, including Sequential, Dense, Flatten, Conv2D, MaxPooling2D, Activation, and Dropout layers. The activation functions were accordingly different based on the multiclass or the binary classification task that we supported. The output model was evaluated by means of accuracy using confusion matrices and classification reports. The model evaluation was performed using an independent test set that had not been used for the training.

### 4.3. Adversarial Imagery Example Generation and Adversarial CNN Model Training

The binary output of the *base CNN model* was used as the main input to craft the different attack algorithms. Several algorithms from the ART tool [15] were used to generate *adversarial imagery examples* and feed them along with the *preprocessed images* to train the *adversarial CNN model*. Table 2 presents the configuration parameters of the the *adversarial CNN model*. In the scenario of the *adversarial CNN model*, the attacked and non-attacked images laying under the same category are labelled with the same original label. The attack algorithms that we used are the fast gradient descent (FGD) attack, DeepFool, NewtonFool, projected gradient descent (PGD), BasicIterative using PGD, SpatialTransformation, SquareAttack, CarliniLIn Method, CarliniL2 Method, and UniversalPerturbation using EAD-elastic-net attacks. The different attack algorithms were used to investigate how effective the attack was and at which level by means of decreasing the model’s accuracy. Decreased accuracy has a direct effect on the model’s robustness, with each attack resulting in adding noise to the images and misclassifying their category.

**Figure 7:**
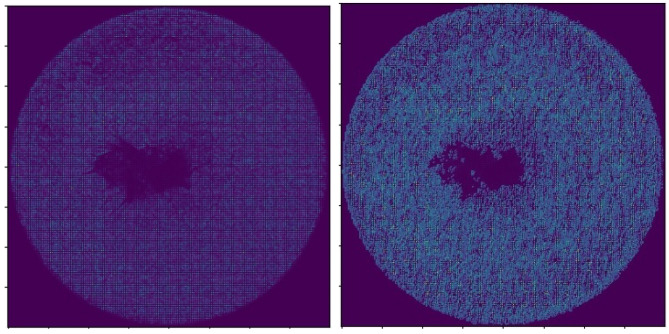
Original vs. FGD attack—*Dataset_1*. The attacked image includes perturbations that were used to distort the original image.

**Figure 8:**
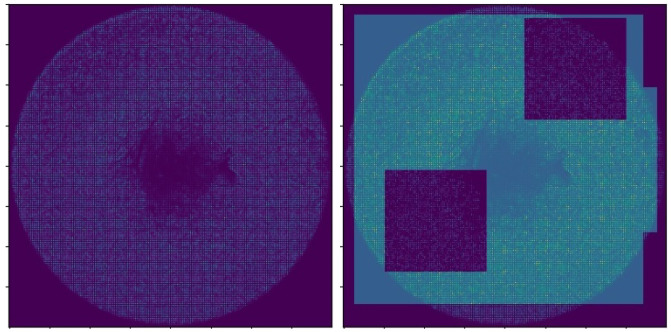
Original vs. square attack—*Dataset_1*. The original image is attacked using localized square-shaped updates at random positions.

Figure 7 shows an example of an original and an adversarial attack by FGD, while Figure 8 depicts an example of an original and an adversarial square attack, both using *Dataset_1*. We have selected two representative examples of non-attacked vs. attacked images. Although in the first example, according to the visual inspection, the effect of the attack is slight, the model’s accuracy is greatly decreased.

At the same time, in the second example, the image cannot be easily distinguished via visual inspection; however, the model’s accuracy is slightly decreased.

**Figure 9:**
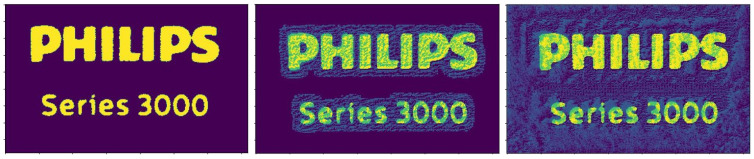
Original (**left**) vs. FGD (**middle**) and PGD (**right**) attacks—*Dataset_2*. FGD attack is slight, while PGD adds a significant amount of noise and color alteration.

A *Dataset_2* example of non-attacked (i.e., original) and attacked images by the fast gradient descent (FGD) and the projected gradient descent (PGD) are depicted in Figure 9. We have selected two representative examples of original vs. attacked images. In the first example, the effect of the FGD attack is slight, while in the second example, the image has more noise and thus can not be easily distinguished. The figure shows that the FGD attack slightly affects the image, while the projected gradient descent highly affects the robustness of the model, resulting in an image that has much noise and color alteration with destroyed resolution.

We performed prediction tasks over the adversarial imagery examples to assess the accuracy of the *base CNN model* compared with the *adversarial CNN model*. A detailed experimental study is presented in Section 5. As an outcome, the *base CNN model* misclassified the adversarial imagery examples, and its accuracy was dramatically decreased. Given this model’s accuracy decrease, we devised a *multiclass discriminator* as a countermeasure to distinguish between original and attacked images and report on each category, as presented in the following section.

### 4.4. Multiclass Discriminator

The *multiclass discriminator* is a model that combines the preprocessed images and the adversarial imagery examples with their labels and is trained to distinguish the non-attacked from the attacked data, i.e., data that an attack algorithm has generated. Table 3 presents the configuration parameters of the *multiclass discriminator*. The scenario of the *multiclass discriminator* labels attacked and original images in different categories. The latter results in multiplying by two (2) categories of the original and the attacked images. In this case, the adversarial imagery examples are labeled with a new label to let the model be trained and predict more accurately if an image lies within an adversarial or an original image category.

### 4.5. Defense Algorithms

**Figure 10:**
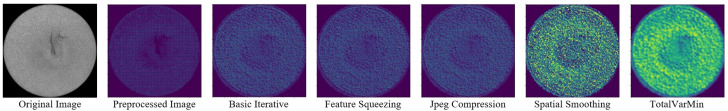
Original and pre-processed images vs. recovered images using the corresponding defenses—*Dataset_1*. The recovered images are used to evaluate a model’s accuracy after the recovery from an attack.

**Figure 11:**
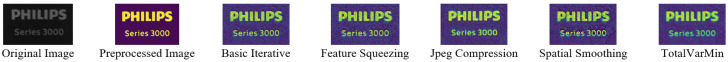
Original and pre-processed vs. recovered images using the corresponding defenses—*Dataset_2*. The recovered images are used to evaluate a model’s accuracy after the recovery from an attack.

*Defense algorithms* are used as another countermeasure to recover and increase the accuracy of a model. We used specific defense algorithms from the ART tool and evaluated the model’s accuracy before and after the recovery from an attack to quantify its score. We used the FeatureSqueezing, JpegCompression, SpatialSmoothing, and TotalVarMin algorithms as defense. We used the test set from the adversarial examples to evaluate how the defense algorithms facilitate recovery from the attacks. We also visualized the results to investigate how the defense algorithms recovered the adversarial examples, as can be seen in Figure 10 and Figure 11.

## 5. Experimental Evaluation

This section presents experimental results on the presented datasets over different attack methods and defense algorithms. Furthermore, we present the experimental results by means of accuracy for the multiclass discriminator. The experimental evaluation is based on the current version of the Adversarial Robustness Toolbox (ART version 1.11.0) [15], Keras (version 2.8.0) [33] and Jupyter Notebooks [34].

The preprocessing module takes care to set the labels of the images. We execute separate experiments for multiclass and binary classification. As far as the multiclass classification is concerned, the images receive the original labels they have, e.g., normal = 1, flow lines = 2 and marks = 3 with respect to *Dataset_1*; and normal = 1, double print = 2 and interrupted print = 3 with respect to *Dataset_2*. We split both datasets into two categories for the binary classification task, e.g., non-attacked = 0 (i.e., usual) and attacked = 1 (i.e., malicious).

### 5.1. Experiments

More than half of the attacks are unable to poison or evade ML algorithms such as SVMs, decision trees or DNNs. Additionally, some attack algorithms are designed and implemented to support only a specific ML/DNN model, library, algorithm and application. In our experiments, we executed different ART attacks to generate the adversarial examples and various defense algorithms to recover the *base CNN model’s* accuracy. Table 4 presents the different algorithms along with their parameters. The set of algorithms both for the attacks and the defenses has been selected based on the criterion to best affect and recover the model’s accuracy according to the data specificities.

Our motivation is to compare and assess the robustness of a DNN model, i.e., the base CNN model, considering its architecture’s specificities, as well as gradient-based attacks, which are more infectious to the backpropagation method used for DNN model training. With these considerations, the reason behind our choice is twofold. On the one hand, our purpose was to be aligned with the existing model used in the actual visual inspection setup described in Section 3.2. In this way, we ensure the applicability of our attacks and defenses to the actual environment being tested. On the other hand, CNNs are the prominent solution used nowadays in the context of computer vision applications. Therefore, our work leaves a wider fingerprint in the AI adversarial literature.

All the following figures visualize the comparison of the model’s accuracy before any attack, on the attacked data, and on the recovered data by the defense. The discriminator’s accuracy has been evaluated on the original and adversarial examples by assessing its predictive capabilities to efficiently identify the correct data category, e.g., for *Dataset_1*, normal = 1, flow lines = 2, and marks = 3. Furthermore, we have used the *K*-fold cross validation method in all the experiments. This method enables us to measure the model’s accuracy in different data samples. Using this method, we also manage to avoid overfitting the data over the model since, before the model training task, the dataset is split into different training and test sets. Specifically, in the experiments conducted, we used *K*-fold to split the dataset into buckets containing all the image categories for both *Dataset_1* and *Dataset_2*.

#### 5.1.1. Binary Classification

**Figure 12:**
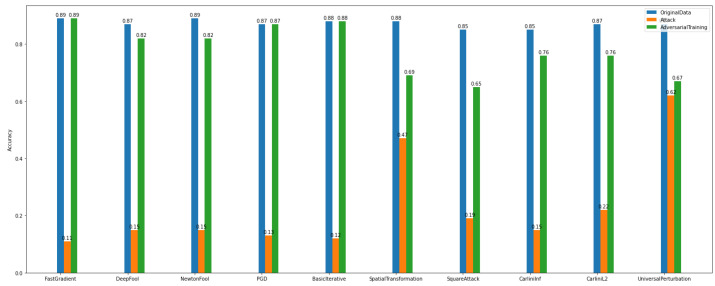
Binary classification—adversarial examples—*Dataset_1*.

In the binary classification task, the data were separated into two categories/classes, i.e., usual = 0 and malicious = 1. This task was evaluated towards the accuracy of the adversarial CNN model and the recovery rate/percentage that the defense algorithms achieved. The algorithms used for attacks are the FastGradient, DeepFool, NewtonFool, PGD, BasicIterative, SpatialTransformation, SquareAttack, CarliniLInf, CarliniL2, and UniversalPerturbation algorithms. We observe in Figure 12 that the class of gradient-based algorithms (e.g., FastGradient, DeepFool, NewtonFool, PGD, BasicIterative, CarliniLInf, and CarliniL2) highly affect the model’s accuracy in *Dataset_1*.

At the same time, according to Figure 12, the creation of adversarial examples has contributed to recovering the impact of the above-mentioned attacks and improving the model’s performance to a satisfactory level.

**Figure 13:**
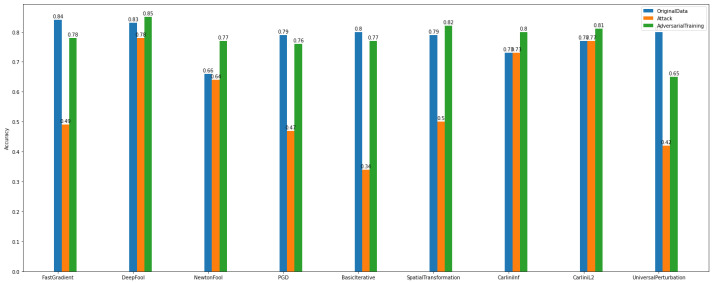
Binary classification—adversarial examples—*Dataset_2*.

Regarding *Dataset_2*, we observe in Figure 13 that the gradient-based attacks have slightly affected the model’s accuracy. In particular, it is obvious that for binary classification on *Dataset_1*, the attack algorithms have a bigger impact on the model’s accuracy than on *Dataset_2*. This proves once again the importance of the specificity of the data.

**Figure 14:**
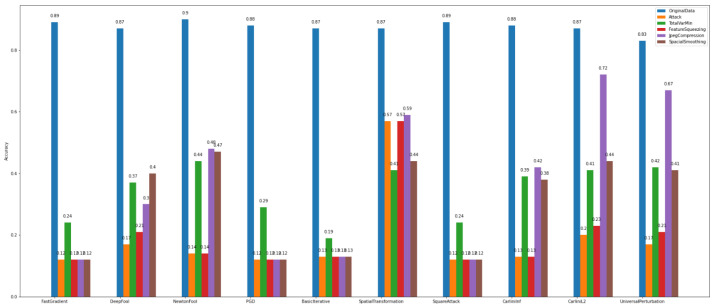
Binary classification—defenses—*Dataset_1*.

Regarding the results of *defense algorithms*, the following figures concentrate the outcomes of the TotalVarMin, FeatureSqueezing, JpegCompression, and SpatialSmoothin algorithms. As far as *Dataset_1* is concerned, in Figure 14, we observe that the SpatialSmoothing and TotalVarMin experience the same recovery rate against the NewtonFool, SpatialTranformation, CarliniLInf, CarliniL2, and UniversalPerturbation attack algorithms.

**Figure 15:**
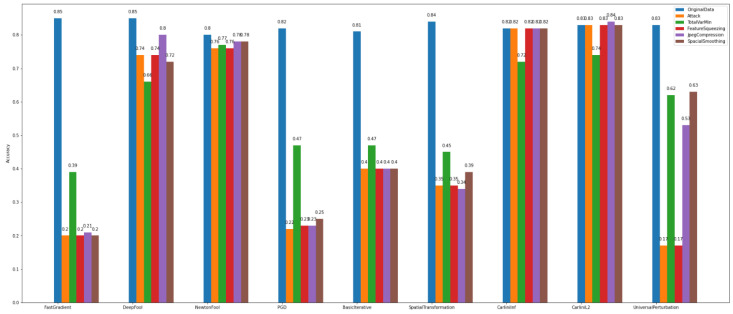
Binary classification—defenses—*Dataset_2*.

Regarding *Dataset_2*, Figure 15 shows that TotalVarMin is the best defense algorithm with the highest recovery rate. A noteworthy aspect is that the Carlini family attackers do not have severe consequences on this dataset, as the model’s accuracy increases as it is trained with more data.

Overall, for binary classification tasks in both datasets (i.e., *Dataset_1* and *Dataset_2*), we observe that training the *base CNN model* with adversarial examples results in making it more robust and recovering its accuracy compared to the *defense algorithms*.

#### 5.1.2. Multiclass Classification

The experimental results are not quantitatively equivalent when conducting the same series of experiments for the multiclass classification task. This is because either more data split over the different categories are required to come up with a robustified multiclass model, or the attacks slightly affect images coming from multiple categories, and therefore this does not affect the model’s accuracy.

**Figure 16:**
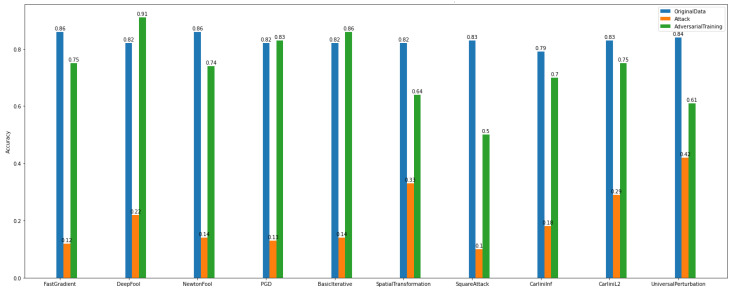
Multiclass classification—adversarial examples—*Dataset_1*.

The *adversarial examples* results, as presented in Figure 16, make the model more vulnerable to attacks. It is obvious that the attack algorithms modify the samples, and as a consequence, the model’s accuracy is decreased.

Specifically, Figure 16 gives information on the performance of adversarial training of *Dataset_1* multiclass case attacks. Again, similarly to the Binary Classification task of the same dataset, by applying adversarial training, the accuracy of the model is recovered from the impact imposed by the gradient-based attacks.

**Figure 17:**
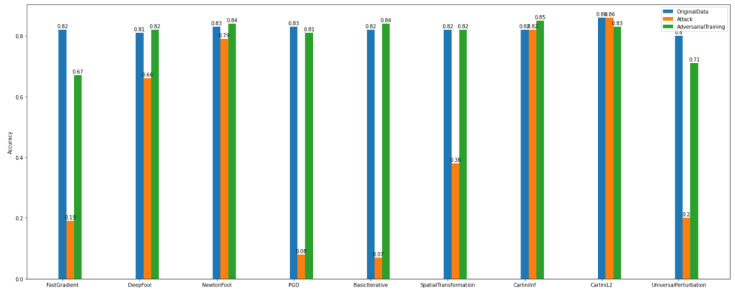
Multiclass Classification—Adversarial Examples—*Dataset_2*.

Figure 17 shows that there are attacks, e.g., Carlini’s, NewtonFool, that do not affect the images. Also, the model injected with adversarial examples has a slight accuracy decrease. On the other hand, Figure 17 for *Dataset_2*, shows that there are attack algorithms that can reduce the model’s accuracy. For instance, Basic Iterative and Project Gradient Descent achieve the lowest performance levels.

**Figure 18:**
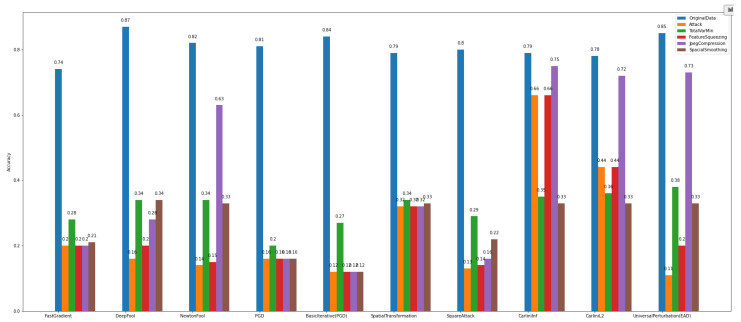
Multiclass classification—defenses—*Dataset_1*.

**Figure 19:**
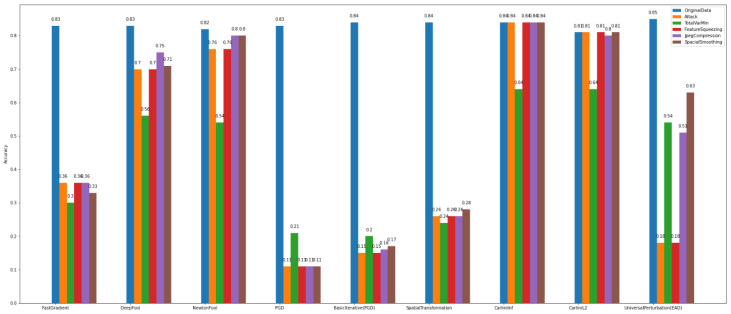
Multi-class classification—defenses—*Dataset_2*.

The *defense algorithm* results, as presented in Figure 18 and Figure 19, show variable recovery rates based on the method. Overall, for *Dataset_1*, the defense algorithm named TotalVarMin appears to achieve a slight increase in accuracy compared to other defense algorithms. More specifically, regarding the attacks FastGradient, DeepFool, PGD, BasicIterative, SpatialTransformation, and SquareAttack, the model’s accuracy shows a measurable increase, where NewtonFool, CarliniLinf, CarliniL2, and UniversalPerturbation do not change the model and thus do not alter its accuracy.

Similarly, the model that is trained over *Dataset_2* has lower accuracy for the same attack algorithms. The defense algorithm TotalVarMin regarding the FastGradient, DeepFool, NewtonFool, SpatialTransformation, CarliniLinf, and CarliniL2 methods has a lower impact on the model’s accuracy.

Upon closer analysis, however, one might note that each attack method can be efficiently treated by at least one or a set of different defense algorithms. Based on the data characteristics and complexity, a careful selection of the best-performing defense algorithm may result in recovering the model’s accuracy at a high rate. At the same time, if an end user looks for a one-size-fits-all solution, adversarial training seems to be the most effective way to recover the overall robustness of the model. The experimental results demonstrate an average increase on the model’s accuracy after augmenting its training with adversarial examples. However, adversarial training is ineffective when we need a clear criterion to distinguish between the original and the adversarial examples because they are both labeled as good/usual samples. This is the reason why we implemented and conducted experiments with the *multiclass discriminator*.

#### 5.1.3. Binary and Multiclass Discriminator

The binary and multiclass discriminator obtains as input the original *N* categories from the datasets. Then, after generating the adversarial imagery examples using one attack algorithm per experiment, another *N* categories are created. The combination of 2*N categories along with their labels as the target variables are used for training the discriminator model in order to classify and predict the right category, i.e., attacked vs. non-attacked and image origin, i.e., normal = 1, flow lines = 2 and marks = 3 with respect to *Dataset_1* and normal = 1, double print = 2 and interrupted print = 3 with respect to *Dataset_2*.

**Figure 20:**
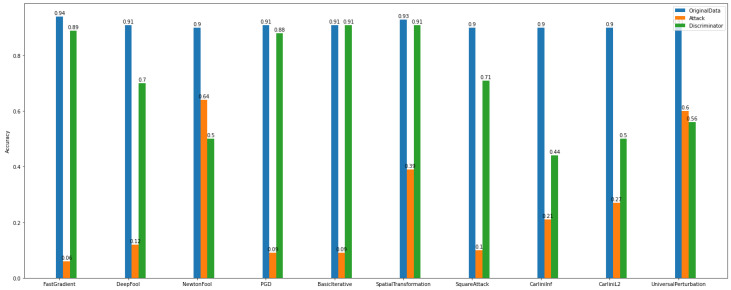
Binary discriminator—*Dataset_1*.

**Figure 21:**
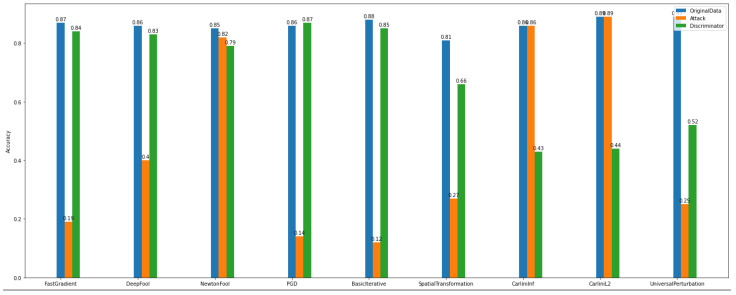
Binary discriminator—*Dataset_2*.

By observing Figure 20 and Figure 21, we can deduce that ART attacks can significantly destroy the model’s performance. For instance, FGD, basic iterative, and PGD attacks have a major influence on the model’s accuracy.

On the other hand, after discriminator implementation and application, the improvement of the model’s performance is unquestionable both in multiclass and binary experiments.

**Figure 22:**
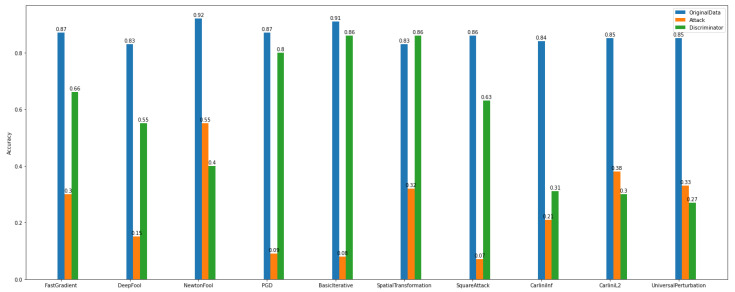
Multi-class discriminator—*Dataset_1*.

Regarding the accuracy of the multiclass discriminator of *Dataset_1* (See Figure 22), the model has high performance. For example, using the PGD attack, the discriminator model can predict and distinguish the differences between the original and the adversarial examples.

**Figure 23:**
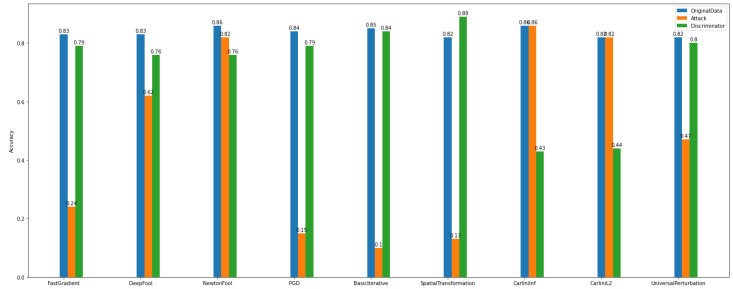
Multi-class discriminator—*Dataset_2*.

In addition, the multiclass and binary discriminator model’s overall behavior seems to follow the same norm. Something worth noticing is that in *Dataset_2*, NewtonFool and the Carlini family attackers do not have a notable impact on the model’s performance (See Figure 23).

Furthermore, after considering the discriminator’s model performance according to the evaluation and plots, the impact of the NewtonFool and the Carlini attacks on the model’s accuracy is high. Therefore, the model’s accuracy is not able to be recovered to any level close to where it was before the attacks.

## 6. Conclusions and Future Work

This work presented a comparative study on adversarial machine learning (AML) attacks on deep neural networks (DNNs), and more specifically, convolutional neural networks (CNNs). In a nutshell, CNNs are prone to adversarial attacks, which present a challenge to safety-critical domains where calibrated, robust, and efficient measures of data uncertainty are crucial.

We introduced a reproducible AI architecture for making CNN image classifiers more robust, which has been evaluated in non-trivial manufacturing environments prone to noise, vibrations, and errors when capturing and transferring data. The proposed architecture’s reproducibility is guaranteed through open-source tools and adversarial and defense algorithms, synthesized and engineered under a systematic approach to guarantee the security and operational assurance of the native systems used in the production lines. In addition, the architecture is composed of modules that robustify and guide the deployment of AML attacks and defense algorithms for recovering the CNN classifiers’ accuracy. That is, new modules can be added to easily extend the architecture’s functionalities and the protection coverage of classifiers used in the underlined systems.

We presented a qualitative study showcasing how the different attacks and defense methods affect the resolution and clarity of the images. This study was further enriched by quantitative experiments measuring the base CNN model’s accuracy in various attack-defense contexts. Overall, it is evident that binary classification results achieve higher accuracy categorizing as usual vs. malicious data, enabling fewer opportunities to uniquely detect from which class the image was originally from. At the same time, despite multiclass classification results achieving lower accuracy, defense algorithms still achieve high recovery rates, enabling the unique prediction of the class of new images.

We may conclude that each case and dataset may require a different defense strategy to be deployed in order to safeguard a baseline model. Different adversarial approaches can be addressed by different defenses, but there is no clear indication that there is a defense strategy that can cover the wide range of attack techniques in an adequate manner. As expected, between the adversarial training and the tested ART defenses, the former seems to be the most effective way to recover the overall model robustness. According to the results of the plots that demonstrate an average accuracy increase after the adversarial training technique, one can observe that it has better performance than the evaluated off-the-shelf defenses offered by ART. Of course, this does not imply that these defenses should not be considered in future experiments, but there is evidence suggesting that, at least for the evaluated datasets, they cannot adequately limit the impact of an adversarial technique. Again, the challenging nature of the problem is that, as advocated by the results, each dataset and each different attack imply the need for the placement of a different defense strategy.

The high success rates of the adversarial attacks against a real-world case, utilizing actual data stemming from a real manufacturing environment, make evident that today’s AI-enabled manufacturing system can become the low-hanging fruits of intelligent attackers. That is, research and innovation need to be fostered in order to design robust architectures for sanitizing the data pipelines of manufacturing environments, filtering out malicious instances and detecting the injection of adversarial examples in the process.

In the near future, we plan to assess the efficacy of the data-centric AI architecture in other application contexts and extend it to other imagery categories with variable features and resolution characteristics.

## Figures and Tables

**Table 1:** Base Convolutional Neural Network (CNN) model configuration parameters.

Multiclass and Binary Classification		
**Dataset**	***Dataset_1***	***Dataset_2***
**Num of Epochs**	15	10
**Batch Size**	30	200
**Input Layer**	(400,400,1)	(220,360,1)
	units: 8	units: 16
	kernel_size: (3,3)	kernel_size: (3,3)
	activation_function: relu	activation_function: relu
	max_pool_size: (2,2)	max_pool_size: (2,2)
	dropout: 0.25	dropout: 0.25
**Output Layer**	activation_function: softmax	activation_function: softmax
**Crossentropy Loss**	categorical and binary	categorical and binary
**Optimizer**	adam	adam

**Table 2:** Adversarial Convolutional Neural Network (CNN) model configuration parameters.

Multiclass and Binary Classification		
**Dataset**	***Dataset_1***	***Dataset_2***
**Num of Epochs**	15	10
**Batch Size**	100	200
**Output Layer**	activation_function: softmax	activation_function: softmax

**Table 3:** Multi-class discriminator configuration parameters.

Multiclass and Binary Classification		
**Dataset**	***Dataset_1***	***Dataset_2***
**Num of Epochs**	25	25
**Batch Size**	100	200
**Output Layer**	activation_function: softmax	activation_function: softmax

**Table 4:** ART attack/defense configuration parameters.

Attack Algorithms	Parameters
FastGradientMethod	norm: np.inf, eps: 1.0, eps_step: 0.1, targeted: False, num_random_init: 0, batch_size: 5, minimal: False
DeepFool	max_iter: 5, epsilon: 1e-6, nb_grads: 10, batch_size: 1, verbose: True
NewtonFool	max_iter: 5, eta: 0.01, batch_size: 1, verbose: True
ProjectGradientDescent	norm: np.inf, eps: 0.3, eps_step: 0.1, max_iter: 10, targeted: False, num_random_init: 0, batch_size: 5, random_eps: False, verbose: True
BasicIterative	eps: 0.3, eps_step: 0.1, max_iter: 5, targeted: False, batch_size: 50
SpatialTransformation	max_translation: 10.0, num_translations: 3, max_rotation: 30.0, num_rotations: 3, verbose: True
SquareAttack	norm: np.inf, max_iter: 100, eps: 0.3, p_init: 0.8, nb_restarts: 1, batch_size: 128, verbose: True
CarliniInf	confidence: 0.0, targeted: False, learning_rate: 0.01, max_iter: 5, max_halving: 5, max_doubling: 5, eps: 0.3, batch_size: 128, verbose: True
CarliniL2	confidence: 0.0, targeted: False, learning_rate: 0.01, binary_search_steps: 10, max_iter: 5, initial_const: 0.01, max_halving: 5, max_doubling: 5, batch_size: 1, verbose: True
UniversalPerturbation	attacker: ead, attacker_params: [max_iter: 5], delta: 0.2, max_iter: 1, eps: 10.0, norm: np.inf, batch_size: 32, verbose: True
DefenseAlgorithms	Parameters
TotalVarMin	prob: 0.3, norm: 2, lamb: 0.5, solver: L-BFGS-B, max_iter: 10, clip_values: None, apply_fit: False, apply_predict: True, verbose: False
FeatureSqueezing	clip_values: [0.0, 1], bit_depth: 8, apply_fit: False, apply_predict: True
JpegCompression	clip_values: [0.0, 1], quality: 50, channels_first: False, apply_fit: True, apply_predict: True, verbose: False
SpatialSmoothing	window_size: 3, channels_first: False, clip_values: None, apply_fit: False, apply_predict: True
